# Longitudinal study of the bacterial and fungal microbiota in the human sinuses reveals seasonal and annual changes in diversity

**DOI:** 10.1038/s41598-019-53975-9

**Published:** 2019-11-22

**Authors:** Brett Wagner Mackenzie, Kevin Chang, Melissa Zoing, Ravi Jain, Michael Hoggard, Kristi Biswas, Richard G. Douglas, Michael W. Taylor

**Affiliations:** 1 School of Medicine, Department of Surgery, The University of Auckland, Auckland, New Zealand; 2 Statistical Consulting Centre, Department of Statistics, The University of Auckland, Auckland, New Zealand; 3 School of Biological Sciences, The University of Auckland, Auckland, New Zealand; 4 Maurice Wilkins Centre for Molecular Biodiscovery, The University of Auckland, Auckland, New Zealand

**Keywords:** Bacteria, Fungi, Microbiome

## Abstract

There is a pressing need for longitudinal studies which examine the stability of the sinonasal microbiota. In this study, we investigated bacterial and fungal community composition of the sinuses of four healthy individuals every month for one year, then once every three months for an additional year to capture seasonal variation. Sequencing of bacterial 16S rRNA genes and fungal ITS2 revealed communities that were mainly dominated by members of *Actinobacteria* and *Basidiomycota*, respectively. We observed overall shifts in both bacterial and fungal community diversity that were attributable to a combination of individual, seasonal and annual changes. The results suggest that each of the subjects possessed a strong bacterial sinonasal signature, but that fungal communities were less subject specific. Differences in fungal and bacterial diversity between subjects, and which OTUs may be correlated with seasonal differences, were investigated. A small core community that persisted throughout the two year sampling period was identified: *Corynebacterium*, *Propionibacterium* and *Staphylococcus*, and one type of fungus, *Malassezia restricta*. It is likely that bacterial and fungal airway microbiomes are dynamic and experience natural shifts in diversity with time. The underlying reasons for these shifts appear to be a combination of changes in environmental climate and host factors.

## Introduction

The sinonasal microbiome is believed to be important for the development and maintenance of mucosal health^1–12^. However, defining the “healthy” microbiome solely in the context of cross-sectional composition may not be reliable, as many of the same microbes can be detected in both healthy and chronically diseased sinuses^8,13–19^. A more holistic definition of the “healthy” sinonasal microbiome should incorporate composition, resilience, resistance, stability, and account for how environmental and host factors can influence these observations^20–28^. While cross-sectional studies have documented inter-personal variations in sinonasal bacteria and fungi^14,15,29^, very few longitudinal studies have tracked intra-personal changes in both fungal and bacterial community composition^25^. There is a pressing need for longitudinal studies which examine the stability of the sinonasal microbiota if we are to understand and anticipate how microbial state shifts influence, and are influenced by, acute and chronic sinonasal diseases.

Several temporal studies have assessed bacterial community stability of the anterior nares, but these results have not been conclusive^21,30–36^. Only one temporal study has assessed the bacterial community of the sinus cavity^35^, although sampling was limited to four times in three weeks, giving no information about seasonal effects and long-term stability. The most comprehensive longitudinal study performed to date of the anterior nares microbiome followed 25 subjects monthly for 15 months^34^. This study employed terminal restriction fragment length polymorphism (T-RFLP) to track changes in bacterial species and concluded that a small core bacterial community was common to all subjects, but that species dynamics over time were found to be person specific. Additionally, a quite extensive change in bacterial community diversity from the winter to summer months was observed. Bacterial community diversity in samples taken during the second winter, however, did not closely resemble that from the first winter sampling point. These results suggested that although seasonal shifts in bacterial community diversity occur, the direction of these shifts might not be predictable and may be due to a number of host and environmental factors^34^.

No longitudinal studies have examined sinonasal fungal and bacterial community diversity and stability in parallel. The extent to which bacterial and fungal members impact the temporal dynamics of the sinonasal microbiome is unknown. One cross-sectional study applied fungal ITS1 and 18S rRNA, as well as bacterial 16S rRNA gene sequencing to examine the variation in microbiome diversity across several body sites, including the anterior nares^37^. The microbiome of the anterior nares was characterised by an abundance of *Malassezia* species, and bacteria from the phylum *Actinobacteria* were negatively correlated with members of the fungal phyla *Basidiomycota* and *Ascomycota*^37^. Another recent cross-sectional study characterised bacteria and fungi in the middle meatuses of control subjects and patients with chronic rhinosinusitis. *Malassezia* species dominated all samples^38^ and season of sampling explained the largest proportion of variation in the mycobiota. Few significant associations between bacteria and fungi were noted.

Little is known about the long-term carriage of bacteria and, especially, fungal species in the sinonasal cavity, and how these communities impact on each other over time. In this study, we examined the temporal composition of bacterial and fungal communities in parallel in a cohort of four healthy adults. We established that these communities are dynamic and may correlate with seasonal changes. Additionally, we identified core bacterial and fungal community members that were present consistently across the two year sampling period, and conducted correlation analyses between bacterial and fungal taxa.

## Results

DNA was extracted from 128 swab samples from four subjects, and the PCR-amplified fungal ITS2 and V3-V4 hypervariable regions of bacterial 16S rRNA genes were sequenced using Illumina MiSeq. Rarefaction of bacterial amplicon data to 1,057 sequences per sample led to the retention of 123 samples, and rarefying fungal amplicon data to 2,706 sequences per sample enabled retention of 127 samples. Sampling time points, self-reported instances of airway-associated illnesses, and antibiotic prescription information are reported in Supplementary Table S1.

Adonis permutational analyses of variance tested the significance and magnitude of contribution due to the combined effect of subject, sampling side, month, season, and year separately for bacterial and fungal communities (Table S2). Between-subject differences in bacterial (48.7%, *p* = 0.001) and fungal (11.3%, *p* = 0.001) communities contributed significantly to overall observed variation. For a given subject, no effect of sampling side was observed. Additionally, differences between seasons and years accounted for a larger proportion of variation for fungal communities (15.3%, *p* = 0.011) than for bacteria (9.9%, *p* = 0.001). The combined effect of months within seasons and years between subjects accounted for the largest proportion of variation in the fungal dataset (19.9%, *p* = 0.001). The inclusion of temporal variables and multiple sampling points drastically reduced the amount of variation unaccounted for in both bacterial and fungal communities (remaining residuals in the final model = 13.8% and 34.5%, respectively).

### Variation in microbial diversity within and between subjects

**Figure 1:**
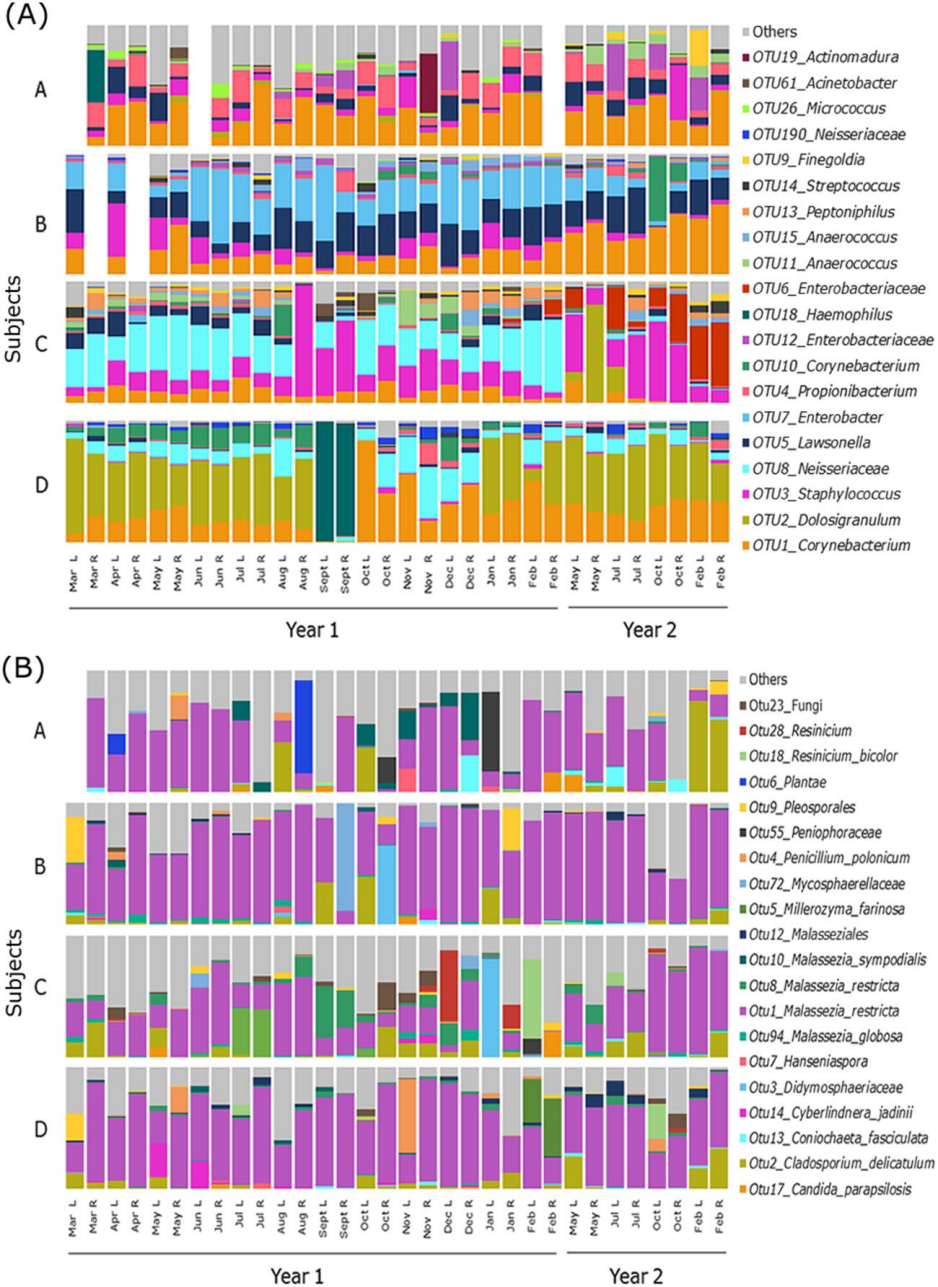
Relative sequence abundances of (**A**) bacterial and (**B**) fungal taxon-assigned OTUs at 97% sequence similarity in the left and right middle meatus swab samples taken from each of the four subjects (**A**–**D**) throughout the two-year study. The 20 most abundant OTUs on average for both bacterial and fungal data are shown, with all other OTUs grouped in “Others”. Missing bars reflect samples that did not pass sequence quality filtering.

Based on average relative abundances, members of the bacterial genera *Corynebacterium* (24.7 ± 16.8% standard deviation), *Dolosigranulum* (10.9 ± 21.4%), and *Staphylococcus* (10.6 ± 14.9%) comprised the largest proportion of sequences in this study, though variation between individual subjects was considerable (Fig. 1A). The number of observed OTUs within each individual varied throughout the two year time period (Figure S1), however no significant differences within subjects between seasons and years were observed. Alpha diversity pairwise comparisons between subjects revealed significant differences in diversity between subjects (Figure S2). Coefficient of variation (CV) values suggested that bacterial communities in the left and right middle meatus sides from all subjects were relatively stable in the number of OTUs over the two year period (all subjects CV values <40%) (Table S3).

Assessment of relative abundance of taxon-assigned fungal OTUs revealed that *Malassezia* (53.9 ± 26.9%), *Cladosporium* (5.96 ± 11.9%), and *Pleosporales* (2.17 ± 7.16%) comprised the majority of fungal sequences in this study (Fig. 1B). A single OTU assigned as *Malassezia restricta* (OTU1) accounted for the largest overall proportion of fungal community diversity in all four subjects (48.9 ± 27.3%), while a tail of diverse, less-abundant fungal OTUs accounted for a substantial proportion of the remaining fungal community diversity (27.6 ± 20.7%). The number of observed fungal-assigned OTUs and their relative abundances within each individual varied throughout the two-year time period (Figure S3) and alpha diversity pairwise comparisons between subjects revealed fewer significant differences between subjects than seen for bacterial community diversity, even though a similar overall number of OTUs was observed (Figure S2). CV values suggested the left and right sides from all subjects were less stable in the number of fungal OTUs (CV values ranged from 23.9% - 87.4%) over the two year period than bacterial OTU counts (28.7% - 38.5%) (Table S3).

**Figure 2:**
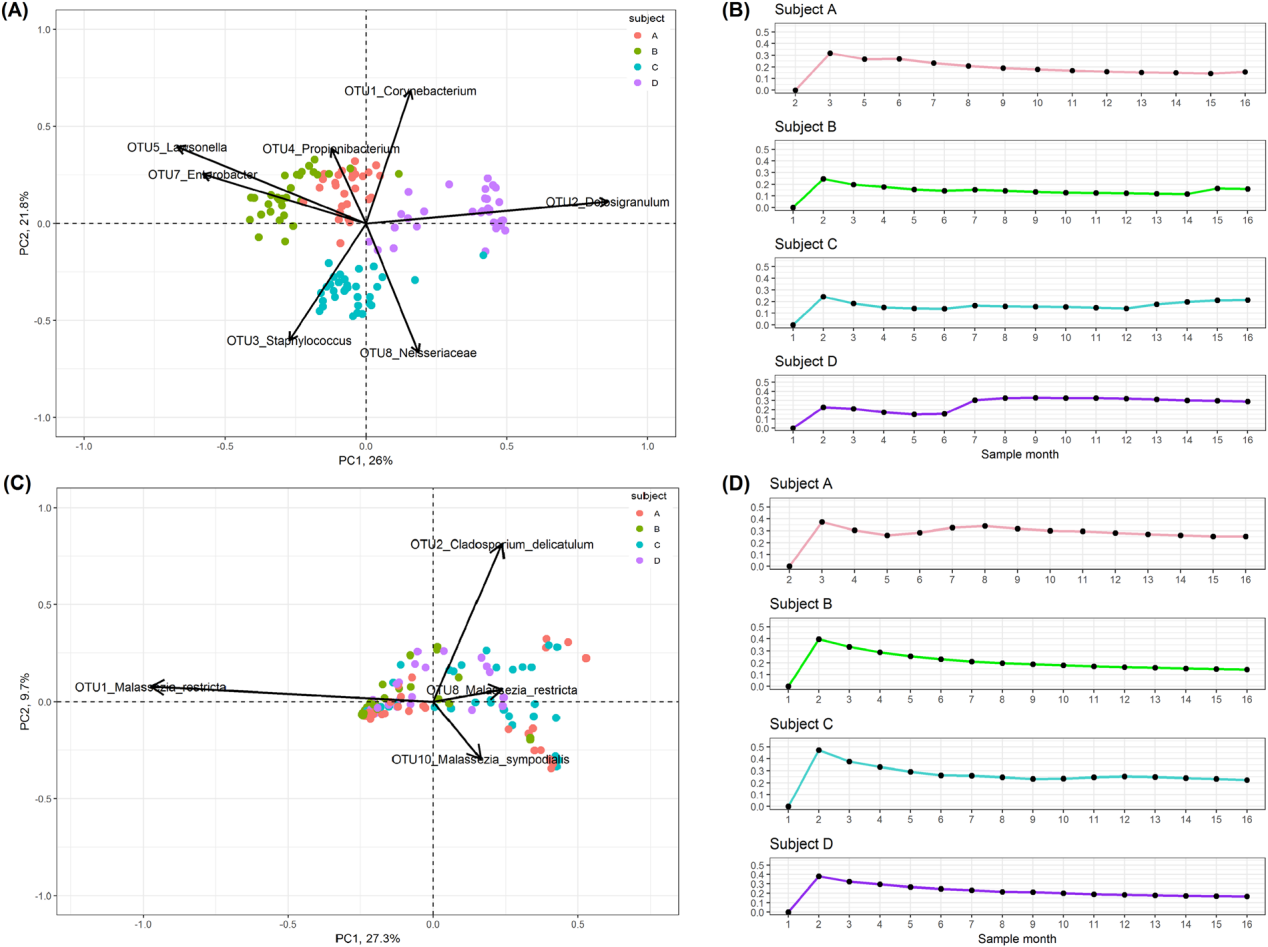
Beta diversity differences are visualised with principal coordinate analysis (PCoA) biplots based on Bray-Curtis dissimilarity metric of the (**A**) bacterial and (**C**) fungal abundance data. Those bacterial and fungal OTUs which are significantly associated with the clustering of samples on the PCoA are overlaid as vectors. The length of the vector indicates the influence of the OTU on the principle component axis. Standard deviations of Bray-Curtis values at different time points from the baseline, showing the changes in the variation of (**B**) bacterial and (**D**) fungal beta diversities over time, for each subject A, B, C and D are visualised as line graphs.

Beta diversity analyses and visualisation of the Bray-Curtis dissimilarity metric in a PCoA plot revealed distinct clustering by subject based on bacterial communities (Fig. 2A). Vectors were overlaid to assess which bacterial OTUs were driving differences between subjects. Each subject could be differentiated from other subjects in the study by a significantly increased relative abundance of specific OTUs. Examination of fungal communities revealed less distinct clustering by subject than bacterial community data, but confirmed that a majority of samples were defined by an abundance of *Malassezia* (Fig. 2C). Temporal variations in each subject’s bacterial and fungal community beta-diversities were evaluated by calculating the standard deviations of Bray-Curtis dissimilarity values at different time points from the baseline then visualised as line graphs (Fig. 2B,D). Notably, a severe shift in the bacterial community of Subject D during September 2015 was observed. This was associated with the onset of acute bacterial sinusitis resulting in a bloom of one OTU associated with *Haemophilus*. A simultaneous shift in the fungal community of Subject D was not observed.

### Bacterial quantification with Droplet Digital™ PCR

Analysis of variance (ANOVA) results revealed that bacterial load within the same subject across different seasons remained relatively stable, as very few significant differences within the same subject across different seasons were reported. Subject C exhibited a significant increase in bacterial load during spring (average number of 16S rRNA gene copies per sample ± standard deviation = 4194 ± 8438) when compared with summer (649 ± 1130, *p* = 0.002) (data not shown).

### Bacterial and fungal core communities

Similar to other human microbiome studies^39–41^, we defined the core sinus community as OTUs that were present in 90–100% of all samples, and then calculated the core communities present in the left and right middle meatus samples from each subject (Table S4, S5), and for all subjects combined. A large number of bacterial and fungal OTUs were transient (present in <20% of all samples), with very few OTUs appearing as persistent colonisers (45–89%) (Figure S4). Slightly different bacterial and fungal core communities between subjects and sides were detected, but the combined core community present in 99% of samples across the entire dataset included three bacterial OTUs (OTU1 *Corynebacterium*, OTU3 *Staphylococcus*, and OTU4 *Propionibacterium*) and one fungal OTU (OTU1 *M. restricta*).

### Seasonal variation in bacterial and fungal communities

A Kruskal-Wallis rank sum test followed by pairwise Dunn’s test between all seasons and years revealed a number of bacterial and fungal OTUs which significantly changed in relative sequence abundance (Table S6). A total of seven bacterial OTUs were associated with at least one significant shift in abundance during the sampling period, whereas 14 fungal OTUs exhibited a significant shift. The bacterial OTU17 (affiliated with the genus *Acidocella*) was associated with a significant increase in relative abundance in all seasons between the first and second sampling years (*p* < 0.05) (Figure S5).

Several fungal OTUs exhibited significant seasonal shifts during both sampling years. For example, OTU13, associated with the fungus *Coniochaeta fasciculata*, increased in relative abundance throughout winter, spring and summer during the first sampling year, then decreased in autumn (the first sample point in year 2). Other fungal OTUs revealed volatile temporal patterns, where periods of very low relative abundance were followed by significant increases in relative abundance.

### Regression analyses of meteorological data with shifts in bacterial and fungal OTUs

Logistic regression modelling of climate data (temperature (°C), rainfall (mm), atmospheric pressure (mbar), and humidity (%)), with those bacterial and fungal OTUs that reported significant seasonal and annual changes identified a number of positive and negative associations (Table 1). The only bacterial OTU to exhibit a relationship with any of the climate variables was OTU93 (*Streptococcus*), which exhibited a positive relationship with atmospheric pressure (*p* = 0.004).

**Table 1:** Generalised linear modelling to fit a logistic regression model of climate data with bacterial and fungal OTUs.

OTU	Variables fitted to model	Range estimate (±95% C.I.)	*p*-value
OTU93 *Streptococcus*	Pressure	8.83–59.8%	*p* = 0.004
OTU21 *Aspergillus penicillioides*	Pressure	(−7.96)–(−61.1)%	*p* = 0.019
	Humidity	(−5.69)–(−68.77)%	*p* = 0.030
OTU75 *Alternaria breviramosa*	Temperature	10.1–457%	*p* = 0.028
OTU2 *Cladosporium delicatulum*	Rainfall	(−4.46)–(−0.31)%	*p* = 0.024
	Humidity	15.6–215%	*p* = 0.011
OTU160 *Verrucocladosporium dirinae*	Pressure	(−77.9)–(−0.64)%	*p* = 0.048
OTU23 *Fungi*	Pressure	0.026–99.0%	*p* = 0.048
OTU61 *Hymenochaetaceae*	Temperature	14.0–253%	*p* = 0.016
OTU3 *Didymosphaeriaceae*	Humidity	5.62–142%	*p* = 0.026

ANOVA tests dictated the probability that climate variables were associated with each OTU, and the nature of the impact was indicated using 95% confidence interval range estimation.

A total of eight fungal OTUs exhibited significant positive or negative relationships with meteorological data. The *C. fasciculata*-affiliated OTU13 had a significant negative relationship with atmospheric pressure (*p* = 0.009). Typically, OTUs were associated with only one of the climate variables, however two fungal OTUs exhibited complex associations with more than one climate variable. Notably, OTU21 *Aspergillus penicillioides* exhibited a negative relationship with both atmospheric pressure and humidity. OTU2 *C. delicatulum* exhibited a negative relationship with rainfall, and a positive relationship with humidity. Interestingly, an increased abundance of this OTU was associated with clustering samples away from those that were dominated by *M. restricta* (OTU1) in the PCoA.

### Correlation of bacterial and fungal OTUs

**Figure 3:**
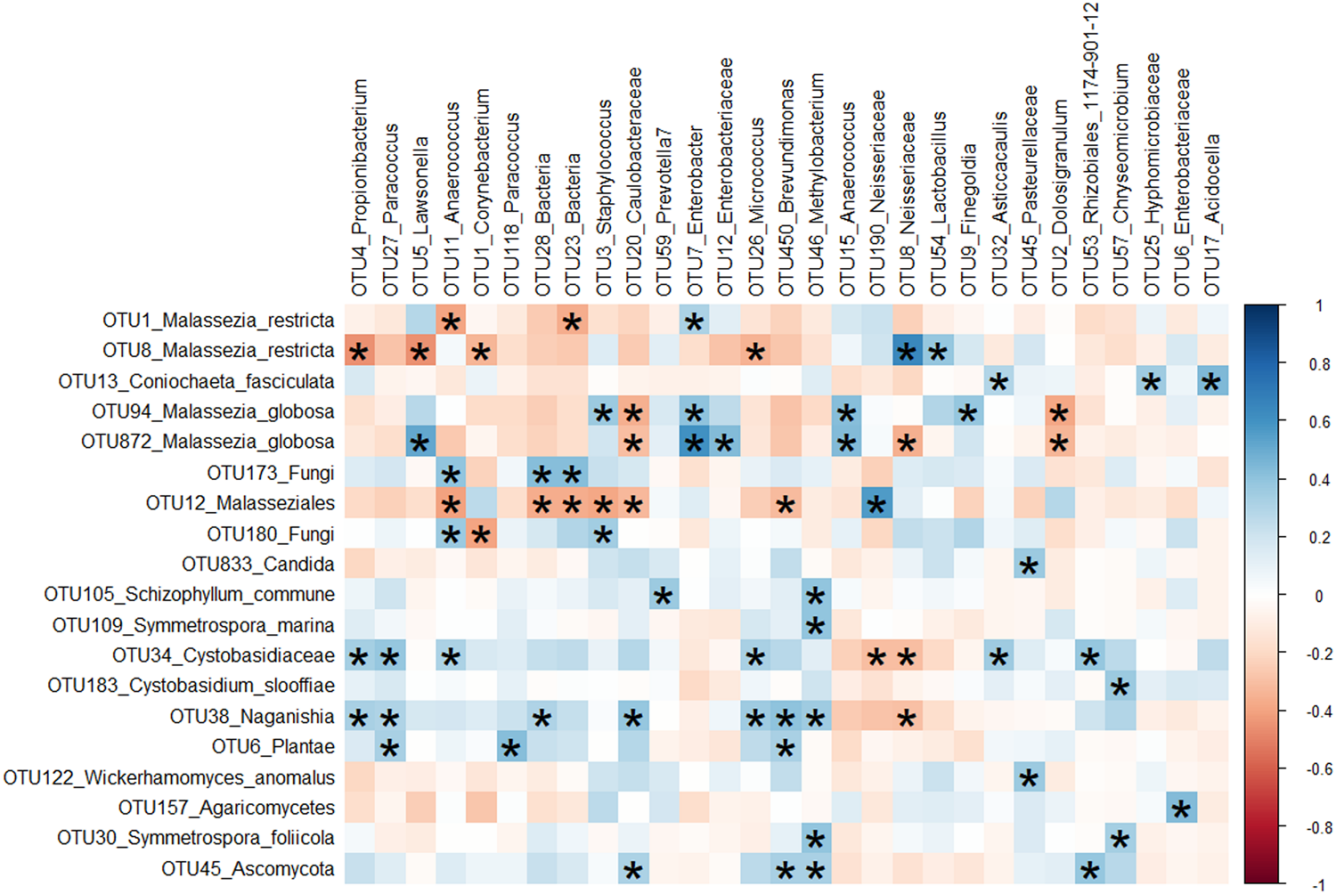
Spearman correlations between bacterial and fungal amplicon data. Both positive (blue) and negative (red) correlations between bacterial and fungal OTUs were calculated. Data were filtered to remove bacterial and fungal OTUs with low prevalence and abundance: OTUs with <0.5% abundance and >99% zeroes across all samples in the dataset were removed. Correlations with at least one absolute strength greater than 35% in each column or row are shown. Correlations are considered significant if *p* < 0.05 after “BH” multi*p*le pairwise comparison correction. Significant correlations are noted with an ‘*’.

To investigate correlations between the alpha and beta diversities of bacterial and fungal communities at the OTU-level, Spearman correlations were calculated. No significant or apparent patterns in the correlation of the number of observed bacterial to fungal OTUs were observed for subjects, seasons or overall samples (Figure S6). There were, however, significant and strong correlations in the abundance and type of bacterial and fungal OTUs across the entire dataset (Fig. 3).

Bacterial OTU4, assigned to the genus *Propionibacterium*, was abundant across all subjects and formed part of the bacterial core community. It was also strongly and significantly negatively correlated with fungal OTU8 *M. restricta*, which may suggest a competitive interaction (though the available data only allow speculation on this point). Bacterial OTU5 (genus *Lawsonella*) was also strongly negatively correlated with fungal OTU8 *M. restricta*, but exhibited a strong positive correlation with *M. globosa* which may suggest these bacterial and fungal OTUs occupy different niches or co-depend on each other.

## Discussion

Dominance of the anterior nares and sinonasal microbiota by bacterial species from the genera *Corynebacterium*, *Staphylococcus*, and *Propionibacterium*^32–34,42^ and several *Malassezia* fungi^16,19,37,43,44^ has been previously reported. These bacteria and fungi have been identified as members of the core nasal microbiome previously^16,34,45^, and the results of our study are consistent with those findings. While OTUs representing *Corynebacterium*, *Staphylococcus*, and *Propionibacterium* were observed as part of the core community, we also noted differences in relative abundances between subjects in carriage of these core OTUs.

High levels of inter-subject variation were observed for bacterial communities in this study. These results, which are supported by previous findings^42^, suggest that an individual sinonasal bacterial fingerprint exists that differentiates subjects from each other. Each individual’s bacterial fingerprint remained relatively stable in both composition and overall load throughout the study. Self-reported instances of viral-mediated colds occurred in all four subjects during August and September of the first sampling year. During this time, no obvious changes in bacterial community structures were noted, as expected for acute viral-associated illnesses. Subject D, however, developed acute bacterial sinusitis (ABS), which was treated effectively with corticosteroids and antibiotics. Interestingly, this subject’s bacterial fingerprint seemed to return to the original profile observed before ABS onset. This observation should be interpreted with caution and validated in future, larger studies that investigate the long-term effect of antibiotics and the recovery of airway bacterial communities from acute infections.

The fungal community from each subject contributed to the overall observed variation, but to a lesser extent than the bacterial communities. High CV values and a lack of subject clustering in PCoA visualisations support the theory that fungal communities are more volatile. This may be due to the observed combination of a high prevalence and abundance of a single fungal OTU (OTU 1, *M. restricta*).

Analyses of shifts between opposite seasons (winter and summer) and ‘shoulder’ seasons (autumn and spring) revealed significant shifts in abundance only in fungal OTUs. While some changes in bacterial OTU abundance were observed chronologically throughout the sampling period (OTUs associated with *Acidocella*, *Hyphomicrobiaceae*, *Asticcacaulis*, and an OTU assigned only as Bacteria), significant changes in abundance were mainly observed when comparing seasons from different years. This suggests that the middle meatus bacterial community is not as susceptible as the fungal community to those factors driving seasonal changes. Rather, these shifts occur over longer periods of time and may be driven by host influences. This supposition is supported by the observed larger proportion of variation attributed to differences between subjects in bacterial communities when compared with fungi.

Unlike the bacterial communities, which had a subject-specific fingerprint, fungal community composition was more influenced by seasonal parameters. The relationship between variation in the diversity and abundance of fungal aerosols with meteorological parameters is well documented^46–52^, and in this study we attempted to elucidate if seasonal changes in climate parameters could be associated with changes in observed fungal composition.

The fungal genus *Cladosporium* was detected throughout the sampling period, which is not surprising given the wide optimal temperature range for growth and likelihood of its year-round presence in New Zealand^53^. The fungal OTU2 associated with *C. delicatulum* exhibited a negative association with rainfall and a positive association with humidity, supporting previous observations that increases in humidity promote sporulation events of this typically indoor air-associated fungus^54^. *Alternaria* species have optimum growth temperatures between 20 and 28 °C, which corresponds to the average autumn and summer temperatures in New Zealand^53^. A significant increase in the relative abundance of *Alternaria breviramosa* during autumn was noted in this study and a positive relationship with temperature was observed. In light of these combined results, future studies focussing on describing sinonasal fungal microbiomes should note sampling season to account for the influence this parameter may have on the results.

Several limitations are associated with this study. First, the small sample size and geographic isolation of this cohort may restrict the generalisability of these results, especially in regards to changes in fungal and bacterial composition with meteorological data. As such, these results should be interpreted with caution. However, the long-term sampling of both the bacterial and fungal composition offers valuable insights into the stability of the sinus microbiota. Although bacterial load was quantified using ddPCR, another limitation of this study is that we did not quantify fungal load throughout the sampling period. A recent study evaluated the effectiveness of a comprehensive primer targeting the fungal 18S rRNA gene for quantification with qPCR^55^ which should be used in future studies. While outside the scope of this study, future longitudinal studies of the human microbiome should also endeavour to measure changes in the functional potential of the microbiome, viral composition and host immunological aspects.

To our knowledge, this is the first study to investigate both fungal and bacterial community composition, as well as bacterial load, in the sinuses across a two-year time period. Microbial communities with high diversity tend to be less stable over time, but complex communities may protect against the establishment of pathogenic organisms^56–58^. Longitudinal studies, such as the one carried out here, increase our understanding that the bacterial and fungal airway microbiomes are dynamic and experience natural shifts in diversity with time. The underlying reasons for these shifts are likely a combination of changes in environmental climate for fungi, and changes within the host for bacterial communities.

## Methods

### Sample collection

Swab samples from four healthy adults, including two male and two female, were collected from the left and right middle meatuses once per month for 12 consecutive months, then once every three months for the second year (representing autumn, winter, spring and summer seasons in the Southern Hemisphere) (Table S1). Potential subjects were excluded based on age <18 years, previous sinus surgery, current/ex-smoker, symptoms of asthma, aspirin sensitivity, and antibiotic and prednisone usage within the six months prior to the first sample collection. Informed, written consent from the patients and ethical approval (NTX/08/12/126) from the New Zealand Health and Disability Ethics Committee was obtained for this study. All research was performed in accordance with relevant guidelines and regulations. Subjects were swabbed at each time point, regardless of antibiotic usage or respiratory health status, resulting in a total of 128 swab samples. Sterile rayon-tipped swabs (Copan, #170KS01) were directed under careful endoscopic guidance to the left and right middle meatus sinuses to sample the surface mucosa. Swabs were collected in duplicate from each side at every time point, placed in 1 mL RNAlater® solution, and stored at −20 °C until DNA extraction.

### DNA collection and target gene amplification

Samples were thawed on ice, and DNA was extracted from pairs of swabs using the Qiagen® AllPrep DNA/RNA Mini Kit (Bio-Strategy Ltd, Auckland, New Zealand) as previously described^59^. Elution Buffer EB (55 µL) was added to the spin column filter and incubated for 5 min before DNA was eluted by centrifuging for 1 min at 11,200 x g. The eluate was centrifuged through the spin column filter a second time to increase DNA concentration. Triplicate negative extractions and PCR amplification of PCR-grade water were performed to test the DNA extraction kits for contamination via PCR. No contamination was observed when PCR products were analysed by agarose gel electrophoresis.

The V3-V4 hypervariable regions of the bacterial 16S rRNA gene and the fungal ITS2 for each sample were amplified. Bacterial gene and fungal ITS2 amplifications and purifications were carried out as described previously^59,60^. Up to 100 ng of template DNA from each sample was amplified, and as many as three PCR replicates were completed for each 16S rRNA bacterial gene or fungal ITS2 amplification. Negative controls comprised PCR-grade water, and *Escherichia coli* or *Candida albicans* genomic DNA were used as positive controls for bacterial or fungal PCRs, respectively.

Bacterial and fungal amplicons were submitted to Auckland Genomics Ltd for library preparation using a dual-indexing approach with Nextera technology and sequencing (2 × 300 bp, paired-end) using Illumina MiSeq.

### Quantification of bacterial 16S rRNA gene copies

Droplet Digital™ PCR (ddPCR) was used to measure absolute quantities of bacterial DNA in samples from corresponding months in the two-year sampling period. Specifically, we investigated the variation in bacterial load across subjects, seasons, and years. Droplet generation, PCR amplification, and QX200 droplet readings were conducted using the QX200™ Droplet Digital PCR System and QuantaSoft™ Software according to the manufacturer’s instructions (Bio-Rad Laboratories). Briefly, the V1-V3 regions of the bacterial 16S rRNA gene were amplified using the primers 8F-341R^61^. Each ddPCR reaction contained 11 µL EvaGreen®, 0.5 µL 10 µM 8 F forward primer, 0.5 µL 10 µM 341 R reverse primer, 9.0 µL of sterile PCR-grade water, and 1.0 µL of sample DNA for a total volume of 22 µL. A positive control of *E. coli* DNA and a negative control of 1X ddPCR buffer with PCR-grade sterile water were included. The thermocycling conditions were as follows: enzyme activation at 95 °C for 5 min, followed by 40 cycles of denaturation at 95 °C for 30 s and annealing/extension at 60 °C for 1 min. A single signal stabilisation step at 4 °C for 5 min then 90 °C for 5 min was carried out. Droplets were analysed using the QuantaSoft™ Software according to the manufacturer’s recommendations. Manual thresholds were set for droplet counts then log_10_ transformed. ANOVA was used to test the three-way interaction between subjects, seasons, and years. The mean value from the same season across years was used for pairwise comparisons within subjects between different seasons followed by Tukey’s *p*-value correction. *P*-value levels <0.05 are considered significant unless otherwise stated.

### Bioinformatic analyses

Bacterial 16S rRNA gene amplicons were processed as described previously^59^. Briefly, amplicons were merged using a minimum merge length of 300 bp, quality filtered, singletons removed, and grouped into operational taxonomic units (OTUs) at 97% sequence similarity using USEARCH^62^. Taxonomic assignment of OTUs against the SILVA 16S rRNA gene database version 128 was performed using the RDP classifier in QIIME version 1.8^63–65^. Human-affiliated OTUs and OTUs that could not be assigned at least to Domain level were removed, then data were rarefied to 1,057 sequences per sample.

Fungal ITS2 amplicons were processed similarly to bacterial sequencing data, except a minimum merge length of 100 bp was applied to account for varying ITS2 sequence lengths. Taxonomic assignment of ITS2 OTUs against the UNITE QIIME release database version 01.12.2017 was performed using the RDP classifier in QIIME version 1.8^63,64,66^. OTUs that could not be assigned at least to Domain level, and OTUs that were human associated, were removed, then data were rarefied to 2,706 sequences per sample as part of ‘core_diversity_analyses.py’ in QIIME version 1.8.

Rarefied bacterial and fungal datasets were used for all downstream analyses. To assess variance in the experimental model from differences in bacterial and fungal communities due to subject, sampling side, month, season, and year, the ‘adonis’ function in the R package ‘vegan’ version 1.11 was applied using the Bray-Curtis dissimilarity matrix^67,68^. Alpha diversity analyses for both amplicon datasets were performed in QIIME version 1.8 to assess the number of OTUs in each sample. Tests for significant differences between the numbers of observed OTUs in each subject were calculated using Dunn’s test with ‘BH’ *p*-value correction, then visualised using box plots generated by ‘ggplot2’ in R version 3.2.5^68,69^. Coefficient of variation (CV) was used to assess alpha diversity stability, measured as the ratio of the standard deviation to the mean number of OTUs in each side over the two year sampling period; higher CV values are assigned to less stable communities.

Data were then filtered to remove bacterial and fungal OTUs with <0.5% abundance. The beta diversity Bray-Curtis distance matrix was generated in ‘vegan’ package and OTU vectors calculated using the ‘envfit’ function. Distances between samples were visualised in a PCoA plot with OTU vectors overlaid using the ‘ggplot2’ package in R version 3.2.5. Temporal variations in each subject’s left side bacterial and fungal community beta-diversities were evaluated by first generating a Bray-Curtis distance matrix for each subject. Then the standard deviations of Bray-Curtis dissimilarity values at different time points from the baseline were calculated and visualised as line graphs using the ‘ggplot2’ package in R version 3.2.5. Core communities (OTUs present in ≥90% of samples) from the left and right sampling sides within each subject were calculated on the full dataset using the ‘compute_core_microbiome.py’ command in QIIME version 1.9 setting a minimum threshold of 0.9 and maximum threshold of 1.0.

### Seasonal changes in microbial communities

Data were filtered to remove bacterial and fungal OTUs with low prevalence and abundance: OTUs with <0.5% abundance and/or >99% zeroes across all samples in the dataset were removed. The Kruskal-Wallis rank sum test was then conducted for all bacterial and fungal OTUs to detect whether a significant difference exists between seasons and years, followed by the non-parametric Dunn’s test to assess specific changes between seasons and years. Dunn’s test does not provide effect sizes, so heat maps were used to visualise changes in relative abundances throughout the sampling period. Heat maps were generated using the ‘pheat map’ and ‘viridis’ packages in R, after log_2_ (x + 1) transformation of data, where x is equal to the sum of OTU counts across all subjects^70,71^.

Meteorological data from Auckland, New Zealand, including monthly average temperature (°C), rainfall (mm), pressure (mbar), and humidity (%) from the period of May 2015 through February 2017 were collated from https://www.worldweatheronline.com/lang/en-nz/auckland-weather-averages/nz.aspx. Model selection using Akaike information criterion (AIC) determined that logistic regression was the best model fit for predicting the relationship between climate variables and bacterial and fungal OTUs. Generalised linear modelling (‘glm’) function in R was applied to fit a logistic regression model of climate data with those bacterial and fungal OTUs which returned significant Kruskal-Wallis *p*-values in any season-year combination (*p* < 0.05). ANOVA tests determined the probability that climate variables were associated with each OTU, and the nature of the impact was indicated using 95% confidence interval range estimation.

### Correlations within and between bacterial and fungal taxa

Correlation of fungal and bacterial OTUs for alpha and beta diversities were calculated in R. Alpha diversity Spearman correlations were calculated using the ‘ggscatter’ function in the R program ‘ggpubr’^72^. Beta diversity Spearman correlations based on Bray-Curtis distances from bacterial and fungal data were calculated in R and visualised using “corrplot”^73^. To be consistent with seasonal calculations, data were filtered to remove bacterial and fungal OTUs with <0.5% abundance and/or >99% zeroes across all samples and timepoints in the dataset. Spearman correlations were then calculated for the remaining bacterial and fungal OTUs. Statistical significance of all correlations was calculated using the function ‘cor.test’ and “BH” correction for multiple pairwise comparisons was applied. A *p*-value <0.05 after “BH” correction was considered significant. Those OTUs with at least one positive or negative correlation greater than 0.35 (±35%) were visualised in a heat map.

## Supplementary information

Supplementary Information

## Data Availability

All sequence data are deposited with NCBI under the BioProject ID number PRJNA464064.
