# Supplementary material for: Longitudinal study of the bacterial and fungal microbiota in the human sinuses reveals seasonal and annual changes in diversity
Source: Sci Rep. 2019 Nov 22;9:17416. doi: 10.1038/s41598-019-53975-9 (PMC6874676; doi:10.1038/s41598-019-53975-9)
Supplement: Supplementary file 1 — Supplementary Information [file 41598_2019_53975_MOESM1_ESM.pdf]

Longitudinal study of the bacterial and fungal microbiota in the human sinuses reveals seasonal and annual changes in diversity

Brett Wagner Mackenzie[1], Kevin Chang[2], Melissa Zoing[1], Ravi Jain[1], Michael Hoggard[3], Kristi Biswas[1], Richard G. Douglas[1], Michael W. Taylor*[3,4]

[1] School of Medicine, Department of Surgery, The University of Auckland, Auckland, New Zealand

[2] Statistical Consulting Centre, Department of Statistics, The University of Auckland, Auckland, New Zealand

[3]School of Biological Sciences, The University of Auckland, Auckland, New Zealand

[4]Maurice Wilkins Centre for Molecular Biodiscovery, The University of Auckland, Auckland, New Zealand

*** Correspondence:**
Michael W. Taylor
mw.taylor@auckland.ac.nz

Supplementary Information

Table S1. Sample collection information throughout the study, including self-reported instances of colds, respiratory illnesses, and medical prescription information. Collection dates are annotated as DD.MM.YYYY.

Subject	Collection number	Collection Date	Swabs collected
A	1	12.03.2015	MM*, 2R, 2L
B	1		MM, 2R, 2L
C	1		MM, 2R, 2L
D	1		MM, 2R, 2L
A	2	24.04.2015	MM, 2R, 2L
B	2		MM, 2R, 2L
C	2		MM, 2R, 2L
D	2		MM, 2R, 2L
A	3	21.05.2015	MM, 2R, 2L
B	3		MM, 2R, 2L
C	3		MM, 2R, 2L
D	3		MM, 2R, 2L
A	4	18.06.2015	MM, 2R, 2L
B	4		MM, 2R, 2L
C	4		MM, 2R, 2L
D	4		MM, 2R, 2L
A	5	16.07.2015	MM, 2R, 2L
B	5		MM, 2R, 2L
C	5		MM, 2R, 2L
D	5		MM, 2R, 2L
A	6	13.08.2015	MM, 2R, 2L
B[1]	6		MM, 2R, 2L
C[1]	6		MM, 2R, 2L
D	6		MM, 2R, 2L
A[1]	7	17.09.2015	MM, 2R, 2L
B[1]	7		MM, 2R, 2L
C[1]	7		MM, 2R, 2L
D[2]	7		MM, 2R, 2L
A	8	29.10.2015	MM, 2R, 2L
B	8		MM, 2R, 2L
C	8		MM, 2R, 2L
D	8		MM, 2R, 2L
A	9	19.11.2015	MM, 2R, 2L
B	9		MM, 2R, 2L
C	9		MM, 2R, 2L
D	9		MM, 2R, 2L
A	10	10.12.2015	MM, 2R, 2L
B	10		MM, 2R, 2L
C	10		MM, 2R, 2L
D	10		MM, 2R, 2L
A	11	29.01.2016	MM, 2R, 2L

B	11		MM, 2R, 2L
C	11		MM, 2R, 2L
D	11		MM, 2R, 2L
A	12	11.02.2016	MM, 2R, 2L
B	12		MM, 2R, 2L
C	12		MM, 2R, 2L
D[3]	12		MM, 2R, 2L
A	13	05.03.2016	MM, 2R, 2L
B	13		MM, 2R, 2L
C	13		MM, 2R, 2L
D	13		MM, 2R, 2L
A	14	26.07.2016	MM, 2R, 2L
B	14		MM, 2R, 2L
C	14		MM, 2R, 2L
D	14		MM, 2R, 2L
A[4]	15	20.10.2016	MM, 2R, 2L
B	15		MM, 2R, 2L
C	15		MM, 2R, 2L
D	15		MM, 2R, 2L
A	16	22.02.2017	MM, 2R, 2L
B	16		MM, 2R, 2L
C	16		MM, 2R, 2L
D	16		MM, 2R, 2L

*MM = Middle meatus sinus, 2R= 2 swab samples from the right middle meatus, 2L= 2 swab samples from the left middle meatus

[1] Subject self-reported instances of a cold

[2] Instance of acute bacterial sinusitis. Subject D was prescribed Amoxicillin 500mg (x15 capsules), (take 1 capsule, orally, 3 times daily), taken for 2 days beginning September 10, 2015. Then, Subject D was prescribed Amoxicillin 500mg + Potassium 125mg (x42 tablets), (take 1 tablet, orally, 3 times daily), taken for 5 days, as well as Prednisone 20mg (x10 tablets), (take 1 tablet once daily, orally), taken for 5 days.

[3] Subject D was prescribed Amoxicillin 500mg + Potassium 125mg (x21 tablets), (take 1 tablet, orally, 3 times daily) on February 4, 2016 for a non-airway related infection.

[4] Subject A was prescribed Flucloxacillin sodium Ca 500 mg (x28 tablets), (take 1 tablet, orally, 4 times daily) on October 6, 2016.

Table S2. Adonis results from (A) Bacterial and (B) Fungal analyses of variance. *P*-values were obtained using 999 permutations under residuals. Terms after ‘Subject’ were added sequentially.

(A)

adonis(formula = bacteria_count_min ~ subject * (sample_year/season)

Factor	Df	SumsOfSqs	MeanSqs	F.Model	R^2	*p*
Subject	3	14.576	4.859	52.629	0.487	0.001
Sample_year	1	0.882	0.882	9.553	0.029	0.001
Sample_year:season	6	1.085	0.181	1.959	0.036	0.001
Subject:sample_year	3	2.004	0.668	7.234	0.067	0.001
Subject:sample_year:season	18	2.977	0.165	1.791	0.099	0.001
Residuals	91	8.401	0.092		0.281	
Total	122	29.924			1.000	

adonis(formula = bacteria_count_min ~ subject * (sample_year/month)

Factor	Df	SumsOfSqs	MeanSqs	F.Model	R^2	*p*
Subject	3	14.576	4.859	69.602	0.487	0.001
Sample_year	1	0.882	0.882	12.634	0.029	0.001
Sample_year:month	14	2.294	0.164	2.348	0.077	0.001
Subject:sample_year	3	2.013	0.671	9.611	0.067	0.001
Subject:sample_year:month	42	6.041	0.144	2.06	0.202	0.001
Residuals	59	4.119	0.070		0.138	
Total	122	29.924			1.000	

adonis(formula = bacteria_count_min ~ subject * (sample_year/season/month)

Factor	Df	SumsOfSqs	MeanSqs	F.Model	R^2	*p*
Subject	3	14.576	4.859	69.602	0.487	0.001
Sample_year	1	0.882	0.882	12.634	0.029	0.001
Sample_year:season	6	1.085	0.181	2.590	0.036	0.001
Subject:sample_year	3	2.004	0.668	9.568	0.067	0.001
Sample_year:season:month	8	1.218	0.152	2.182	0.041	0.001
Subject:sample_year:season	18	2.960	0.164	2.356	0.099	0.001
Subject:sample_year:season:month	24	3.081	0.128	1.839	0.103	0.001
Residuals	59	4.119	0.070		0.138	
Total	122	29.924			1.000	

(B)

adonis(formula = fungi_count_min ~ subject * (sample_year/season)

Factor	Df	SumsOfSqs	MeanSqs	F.Model	R^2	*p*
Subject	3	3.004	1.001	5.703	0.113	0.001
Sample_year	1	0.348	0.348	1.982	0.013	0.036
Sample_year:season	6	1.793	0.299	1.702	0.067	0.001
Subject:sample_year	3	0.722	0.241	1.370	0.027	0.083
Subject:sample_year:season	18	4.108	0.228	1.300	0.154	0.011
Residuals	95	16.682	0.176		0.626	
Total	126	26.657			1.000	

adonis(formula = fungi_count_min ~ subject * (sample_year/month))

Factor	Df	SumsOfSqs	MeanSqs	F.Model	R^2	*p*
Subject	3	3.004	1.001	6.859	0.113	0.001
Sample_year	1	0.348	0.348	2.384	0.013	0.012
Sample_year:month	14	3.991	0.285	1.953	0.150	0.001
Subject:sample_year	3	0.716	0.239	1.636	0.027	0.025
Subject:sample_year:month	42	9.401	0.224	1.533	0.353	0.001
Residuals	63	9.197	0.146		0.345	
Total	126	26.657			1.000	

adonis(formula = fungi_count_min ~ subject * (sample_year/season/month))

Factor	Df	SumsOfSqs	MeanSqs	F.Model	R^2	*p*
Subject	3	3.004	1.001	6.859	0.113	0.001
Sample_year	1	0.348	0.348	2.384	0.013	0.015
Sample_year:season	6	1.793	0.299	2.047	0.067	0.002
Subject:sample_year	3	0.722	0.241	1.648	0.027	0.027
Sample_year:season:month	8	2.192	0.274	1.877	0.082	0.001
Subject:sample_year:season	18	4.089	0.227	1.556	0.153	0.001
Subject:sample_year:season:month	24	5.311	0.221	1.516	0.199	0.001
Residuals	63	9.197	0.146		0.345	
Total	126	26.657			1.000	

Table S3. Coefficient of variation (CV) values as a measure of (A) bacterial and (B) fungal community alpha diversity stability in the left and right sinuses of each subject. CV values were calculated by dividing the standard deviation by the mean number of OTUs across the sampling period for each subject, then multiplying by 100.

(A)

Subject, Side	Average #OTUs ± Standard deviation	Coefficient of Variation
A, Left	51 ± 14.63	28.68%
A, Right	52.27 ± 20.12	38.50%
B, Left	27.25 ± 8.17	29.78%
B, Right	26.86 ± 10.23	38.12%
C, Left	36.19 ± 11.17	30.87%
C, Right	34.75 ±10.12	29.13%
D, Left	19.38 ± 5.69	29.37%
D, Right	20.94 ± 7.11	33.97%

(B)

Subject, Side	Average #OTUs ± Standard deviation	Coefficient of Variation
A, Left	20.33 ± 17.78	87.42%
A, Right	17.00 ± 5.06	29.76%
B, Left	19.31 ± 9.26	47.97%
B, Right	14.31 ± 4.17	29.17%
C, Left	26.31 ± 6.29	23.90%
C, Right	30.00 ± 8.93	29.76%
D, Left	22.38 ± 9.97	44.54%
D, Right	24.38 ± 9.13	37.45%

Table S4. Core bacterial communities in (A) the left and right middle meatus sides of each subject over the two-year sampling period, and (B) with all subjects left and right sides combined. Core communities are defined as permanent OTUs that are present in at least 90% of all samples within that group.

(A)

SUBJECT, SIDE	90%	100%
**SUBJECT A, LEFT**	OTU 4, *Propionibacterium* sp.	OTU 4, *Propionibacterium* sp.
	OTU 5, *Lawsonella* sp.	OTU 5, *Lawsonella* sp.
	OTU 1, *Corynebacterium* sp.	OTU 1, *Corynebacterium* sp.
	OTU 3, *Staphylococcus* sp.	OTU 3, *Staphylococcus* sp.
**SUBJECT A, RIGHT**	OTU 4, *Propionibacterium* sp.	OTU 4, *Propionibacterium* sp.
	OTU 5, *Lawsonella* sp.	
	OTU 11, *Anaerococcus* sp.	
	OTU 1, *Corynebacterium* sp.	OTU 1, *Corynebacterium* sp.
	OTU 23, Bacteria	
	OTU 3, *Staphylococcus* sp.	OTU 3, *Staphylococcus* sp.
**SUBJECT B, LEFT**	OTU 4, *Propionibacterium* sp.	OTU 4, *Propionibacterium* sp.
	OTU 5, *Lawsonella* sp.	OTU 5, *Lawsonella* sp.
	OTU 1, *Corynebacterium* sp.	OTU 1, *Corynebacterium* sp.
	OTU 3, *Staphylococcus* sp.	OTU 3, *Staphylococcus* sp.
	OTU 7, *Enterobacter* sp.	OTU 7, *Enterobacter* sp.
	OTU 12, *Enterobacteriaceae*	OTU 12, *Enterobacteriaceae*
**SUBJECT B, RIGHT**	OTU 4, *Propionibacterium* sp.	OTU 4, *Propionibacterium* sp.
	OTU 5, *Lawsonella* sp.	OTU 5, *Lawsonella* sp.
	OTU 1, *Corynebacterium* sp.	OTU 1, *Corynebacterium* sp.
	OTU 3, *Staphylococcus* sp.	OTU 3, *Staphylococcus* sp.
	OTU 7, *Enterobacter* sp.	OTU 7, *Enterobacter* sp.
	OTU 12, *Enterobacteriaceae*	OTU 12, *Enterobacteriaceae*
	OTU 15, *Anaerococcus* sp.	OTU 15, *Anaerococcus* sp.
**SUBJECT C, LEFT**	OTU 4, *Propionibacterium* sp.	OTU 4, *Propionibacterium* sp.
	OTU 1, *Corynebacterium* sp.	OTU 1, *Corynebacterium* sp.
	OTU 3, *Staphylococcus* sp.	OTU 3, *Staphylococcus* sp.
	OTU 8, *Neisseriaceae*	OTU 8, *Neisseriaceae*
	OTU 9, *Finegoldia* sp.	
**SUBJECT C, RIGHT**	OTU 4, *Propionibacterium* sp.	OTU 4, *Propionibacterium* sp.
	OTU 5, *Lawsonella* sp.	
	OTU 1, *Corynebacterium* sp.	
	OTU 3, *Staphylococcus* sp.	OTU 3, *Staphylococcus* sp.
	OTU 15, *Anaerococcus* sp.	
	OTU 14, *Streptococcus* sp.	
	OTU 8, *Neisseriaceae*	OTU 8, *Neisseriaceae*
	OTU 13, *Peptoniphilus* sp.	OTU 13, *Peptoniphilus* sp.
	OTU 9, *Finegoldia* sp.	

**SUBJECT D, LEFT**	OTU 4, *Propionibacterium* sp.	
	OTU 1, *Corynebacterium* sp.	OTU 1, *Corynebacterium* sp.
	OTU 190, *Neisseriaceae*	OTU 190, *Neisseriaceae*
	OTU 8, *Neisseriaceae*	OTU 8, *Neisseriaceae*
**SUBJECT D, RIGHT**	OTU 4, *Propionibacterium* sp.	OTU 4, *Propionibacterium* sp.
	OTU 5, *Lawsonella* sp.	OTU 5, *Lawsonella* sp.
	OTU 1, *Corynebacterium* sp.	OTU 1, *Corynebacterium* sp.
	OTU 3, *Staphylococcus* sp.	OTU 3, *Staphylococcus* sp.
	OTU 190, *Neisseriaceae*	
	OTU 8, *Neisseriaceae*	OTU 8, *Neisseriaceae*

(B)		
**SUBJECTS COMBINED, LEFT**	OTU 4, *Propionibacterium* sp.	
	OTU 5, *Lawsonella* sp.	
	OTU 1, *Corynebacterium* sp.	OTU 1, *Corynebacterium* sp.
	OTU 3, *Staphylococcus* sp.	
**SUBJECTS COMBINED, RIGHT**	OTU 4, *Propionibacterium* sp.	OTU 4, *Propionibacterium* sp.
	OTU 5, *Lawsonella* sp.	
	OTU 1, *Corynebacterium* sp.	
	OTU 3, *Staphylococcus* sp.	OTU 3, *Staphylococcus* sp.

Table S5. Core fungal communities in (A) the left and right middle meatus sides of each subject over the two-year sampling period, and (B) with all subjects left and right sides combined. Core communities are defined as permanent OTUs that are present in at least 90% of all samples within that group.

(A)

SUBJECT, SIDE	90%	100%
**SUBJECT A, LEFT**	OTU 1, *Malassezia restricta*	OTU 1, *Malassezia restricta*
**SUBJECT A, RIGHT**	OTU 1, *Malassezia restricta*	OTU 1, *Malassezia restricta*
**SUBJECT B, LEFT**	OTU 1, *Malassezia restricta*	OTU 1, *Malassezia restricta*
**SUBJECT B, RIGHT**	OTU 1, *Malassezia restricta*<br>OTU 94, *Malassezia globosa*	OTU 1, *Malassezia restricta*
**SUBJECT C, LEFT**	OTU 1, *Malassezia restricta*<br>OTU 8, *Malassezia restricta*<br>OTU 173, Fungi	OTU 1, *Malassezia restricta*<br>OTU 8, *Malassezia restricta*
**SUBJECT C, RIGHT**	OTU 1, *Malassezia restricta*	OTU 1, *Malassezia restricta*
**SUBJECT D, LEFT**	OTU 1, *Malassezia restricta*<br>OTU 8, *Malassezia restricta*	OTU 1, *Malassezia restricta*
**SUBJECT D, RIGHT**	OTU 1, *Malassezia restricta*	OTU 1, *Malassezia restricta*
(B)		
**SUBJECTS COMBINED, LEFT**	OTU 1, *Malassezia restricta*	OTU 1, *Malassezia restricta*
**SUBJECTS COMBINED, RIGHT**	OTU 1, *Malassezia restricta*	OTU 1, *Malassezia restricta*

Table S6. Evaluation of changes within bacterial and fungal OTUs across seasons and years. (A) Chronologically across the sampling period, (B) Between season years, and (C) the remaining pairwise comparisons of all seasons within and between years. If a Kruskal-Wallis rank sum test generated overall *p*-values that indicated a significant difference existed at least once throughout the sampling period, then pairwise comparisons were made between seasons and years using Dunn's test. *P*-values < 0.05 were considered significant.

(A)

**Bacterial OTUs**	**Overall**	**autumn_year_1 - winter_year_1**	**spring_year_1 - winter_year_1**	**spring_year_1 - summer_year_1**	**autumn_year_2 - summer_year_1**	**autumn_year_2 - winter_year_2**	**spring_year_2 - winter_year_2**	**spring_year_2 - summer_year_2**
OTU17, *Acidocella*	**9.80E-15**	–	–	–	**1.68E-04**	–	–	–
OTU25, *Hyphomicrobiaceae*	**6.27E-06**	–	–	–	**0.012**	–	**0.007**	–
OTU32, *Asticcacaulis*	**1.75E-04**	–	–	–	–	–	**0.047**	–
OTU28, *Bacteria*	**0.03**	–	–	–	–	–	–	–
OTU35, *Alloprevotella*	**0.03**	–	–	–	–	–	–	–
OTU93, *Streptococcus*	**0.04**	–	–	–	–	–	–	–
OTU2, *Dolosigranulum*	**0.04**	–	–	–	–	–	–	–

**Fungal OTUs**	**Overall**	**autumn_year_1 - winter_year_1**	**spring_year_1 - winter_year_1**	**spring_year_1 - summer_year_1**	**autumn_year_2 - summer_year_1**	**autumn_year_2 - winter_year_2**	**spring_year_2 - winter_year_2**	**spring_year_2 - summer_year_2**
OTU13, *Coniochaeta fasciculata*	**1.89E-06**	–	**0.005**	**0.031**	**0.004**	–	–	–
OTU64, *Toxicocladosporium strelitziae*	**1.16E-04**	–	–	–	**1.24E-05**	**0.017**	–	–
OTU862, *Rhodotorula diobovata*	**0.002**	–	–	–	–	–	**0.015**	–
OTU21, *Aspergillus penicillioides*	**0.004**	**0.009**	–	–	–	–	–	–
OTU75, *Alternaria breviramosa*	**0.005**	**0.007**	–	–	–	–	–	–
OTU2, *Cladosporium delicatulum*	**0.006**	–	–	–	–	–	–	**0.001**
OTU160, *Verrucocladosporium dirinae*	**0.012**	–	–	–	**0.010**	**0.011**	–	–
OTU23, *Fungi*	**0.017**	–	**0.037**	**0.039**	–	–	–	–
OTU158, *Poaceae*	**0.021**	–	–	–	–	**0.035**	–	–

OTU61, *Hymenochaetaceae*	**0.021**	–	–	**0.047**	**0.039**	–	–	–
OTU235, *Scytinostroma*	**0.046**	–	–	–	–	–	–	–
OTU383, *Plantae*	**0.046**	–	–	–	–	–	–	**0.031**
OTU183, *Cystobasidium slooffiae*	**0.047**	–	–	–	–	–	–	–
OTU3, *Didymosphaeriaceae*	**0.049**	–	–	–	–	–	–	–

(B)

**Bacterial OTUs**	**Overall**	**autumn_year_1 - autumn_year_2**	**winter_year_1 - winter_year_2**	**spring_year_1 - spring_year_2**	**summer_year_1 - summer_year_2**
OTU17, *Acidocella*	**9.80E-15**	**1.56E-04**	**1.80E-04**	**2.80E-05**	**9.48E-08**
OTU25, *Hyphomicrobiaceae*	**6.27E-06**	**0.005**	**1.07E-04**	–	**0.011**
OTU32, *Asticcacaulis*	**1.75E-04**	–	**3.66E-04**	–	–
OTU28, *Bacteria*	**0.029**	–	–	–	–
OTU35, *Alloprevotella*	**0.029**	–	–	–	–
OTU93, *Streptococcus*	**0.041**	–	–	**0.034**	–
OTU2, *Dolosigranulum*	**0.044**	–	–	–	–

**Fungal OTUs**	**Overall**	**autumn_year_1 - autumn_year_2**	**winter_year_1 - winter_year_2**	**spring_year_1 - spring_year_2**	**summer_year_1 - summer_year_2**
OTU13, *Coniochaeta fasciculata*	**1.89E-06**	**0.004**	**0.001**	–	**0.004**
OTU64, *Toxicocladosporium strelitziae*	**1.16E-04**	**8.31E-05**	–	–	–
OTU862, *Rhodotorula diobovata*	**0.002**	–	–	**0.005**	**0.009**
OTU21, *Aspergillus penicillioides*	**0.004**	–	–	–	–
OTU75, *Alternaria breviramosa*	**0.005**	–	–	–	–
OTU2, *Cladosporium delicatulum*	**0.006**	–	–	–	**0.004**
OTU160, *Verrucocladosporium dirinae*	**0.012**	**0.002**	–	–	–

OTU23, *Fungi*	**0.017**	–	–	–	–
OTU158, *Poaceae*	**0.021**	–	–	–	–
OTU61, *Hymenochaetaceae*	**0.021**	–	–	–	–
OTU235, *Scytinostroma*	**0.046**	–	–	–	–
OTU383, *Plantae*	**0.046**	–	–	–	–
OTU183, *Cystobasidium slooffiae*	**0.047**	–	**0.049**	–	–
OTU3, *Didymosphaeriaceae*	**0.049**	–	–	–	–

(C)

**Bacterial OTUs**	**autumn_year_1 - spring_year_1**	**autumn_year_2 - spring_year_1**	**autumn_year_1 - spring_year_2**	**autumn_year_2 - spring_year_2**	**autumn_year_1 - summer_year_1**	**spring_year_2 - summer_year_1**	**autumn_year_1 - summer_year_2**	**autumn_year_2 - summer_year_2**	**spring_year_1 - summer_year_2**	**autumn_year_2 - winter_year_1**	**spring_year_1 - winter_year_1**	**spring_year_2 - winter_year_1**	**summer_year_1 - winter_year_1**	**summer_year_2 - winter_year_1**	**autumn_year_1 - winter_year_2**	**spring_year_1 - winter_year_2**	**summer_year_1 - winter_year_2**	**summer_year_2 - winter_year_2**
OTU17, *Acidocella*	–	**1.53E-04**	**2.51E-05**	–	–	**2.93E-05**	**6.32E-08**	–	**1.30E-07**	**3.59E-04**	–	**6.54E-05**	–	**1.85E-07**	**8.95E-05**	**9.04E-05**	**9.84E-05**	–
OTU25, *Hyphomicrobiaceae*	–	–	–	–	–	–	**0.004**	–	–	**0.008**	–	–	–	**0.007**	**5.88E-05**	**0.002**	**1.94E-04**	–
OTU32, *Asticcacaulis*	–	**0.050**	–	–	–	–	–	–	–	–	–	–	–	**0.049**	**0.001**	**4.02E-04**	**3.31E-04**	–
OTU28, *Bacteria*	–	–	–	–	–	–	**0.030**	–	–	–	–	–	–	–	–	–	–	–
OTU35, *Alloprevotella*	–	–	–	–	–	–	–	–	–	–	–	–	–	–	–	–	–	–
OTU93, *Streptococcus*	–	–	–	–	–	–	–	–	–	–	–	–	–	–	–	**0.043**	–	–
OTU2, *Dolosigranulum*	–	–	–	–	–	–	–	–	–	–	–	–	–	–	–	–	–	–

Fungal OTUs	autumn_year_1 - spring_year_1	autumn_year_2 - spring_year_1	autumn_year_1 - spring_year_2	autumn_year_2 - spring_year_2	autumn_year_1 - summer_year_1	spring_year_2 - summer_year_1	autumn_year_1 - summer_year_2	autumn_year_2 - summer_year_2	spring_year_1 - summer_year_2	autumn_year_2 - winter_year_1	spring_year_1 - winter_year_1	spring_year_2 - winter_year_1	summer_year_1 - winter_year_1	summer_year_2 - winter_year_1	autumn_year_1 - winter_year_2	spring_year_1 - winter_year_2	summer_year_1 - winter_year_2	summer_year_2 - winter_year_2
OTU13, *Coniochaeta fasciculata*	**0.031**	–	**0.030**	–	–	**0.031**	**0.004**	–	–	**0.002**	**0.005**	**0.010**	–	**0.002**	**0.002**	–	**0.002**	–
OTU64, *Toxicocladosporium strelitziae*	–	**8.28E-06**	–	**2.24E-04**	–	–	–	**2.68E-04**	–	**2.48E-05**	–	–	–	–	–	–	–	–
OTU862, *Rhodotorula diobovata*	–	–	**0.016**	**0.014**	–	**0.006**	**0.016**	**0.014**	**0.006**	–	–	**0.007**	–	**0.019**	–	–	–	**0.015**
OTU21, *Aspergillus penicillioides*	–	–	**0.047**	–	–	–	–	–	–	–	–	–	**0.017**	–	–	–	–	–
OTU75, Alternaria breviramosa	**0.002**	–	**0.031**	–	**0.004**	–	**0.037**	–	–	–	–	–	–	–	**0.047**	–	–	–
OTU2, *Cladosporium delicatulum*	–	–	–	–	–	–	**0.029**	–	**0.007**	–	–	–	–	**0.002**	–	–	–	**0.029**
OTU160, *Verrucocladosporium dirinae*	–	**0.003**	–	–	–	–	–	**0.009**	–	**0.005**	–	–	–	–	–	–	–	–
OTU23, *Fungi*	–	–	–	**0.047**	–	–	–	–	–	–	**0.037**	**0.050**	–	–	–	–	–	–
OTU158, *Poaceae*	–	–	–	**0.026**	–	–	–	–	–	**0.046**	–	–	–	–	–	–	–	–
OTU61, *Hymenochaetaceae*	–	–	–	–	**0.036**	**0.047**	–	–	–	–	–	–	**0.010**	–	–	–	–	–
OTU235, *Scytinostroma*	–	–	–	–	–	–	–	–	–	–	–	–	–	–	–	–	–	–
OTU383, *Plantae*	–	–	–	–	–	–	**0.020**	**0.025**	**0.034**	–	–	–	–	–	–	–	–	**0.041**

OTU183, *Cystobasidium slooffiae*	–	–	–	–	–	–	–	–	–	–	–	–	–	–	**0.017**	**0.025**	–	–
OTU3, *Didymosphaeriaceae*	–	–	–	–	–	–	–	–	–	–	–	–	–	–	–	–	–	–

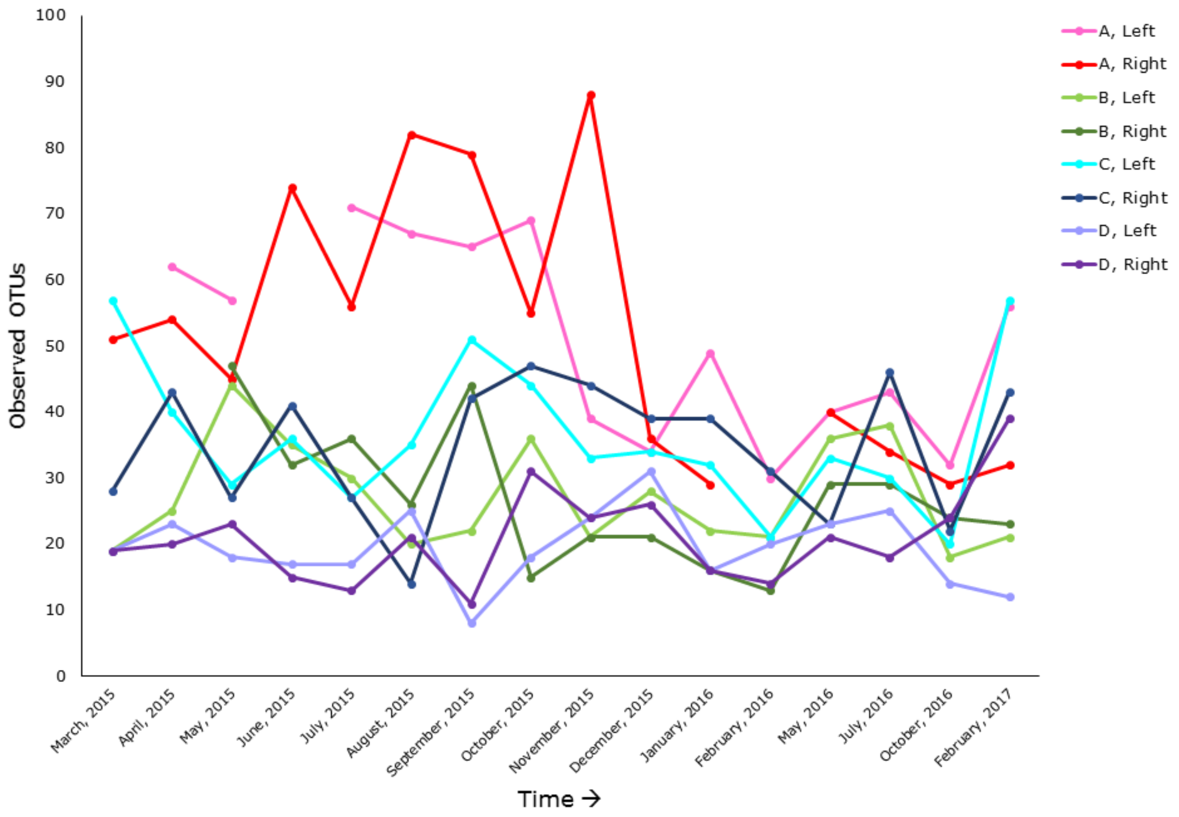

Figure S1. The number of observed bacterial OTUs at 97% sequence similarity from the left and right sinuses of each individual throughout the two-year time period visualised in a line-graph.

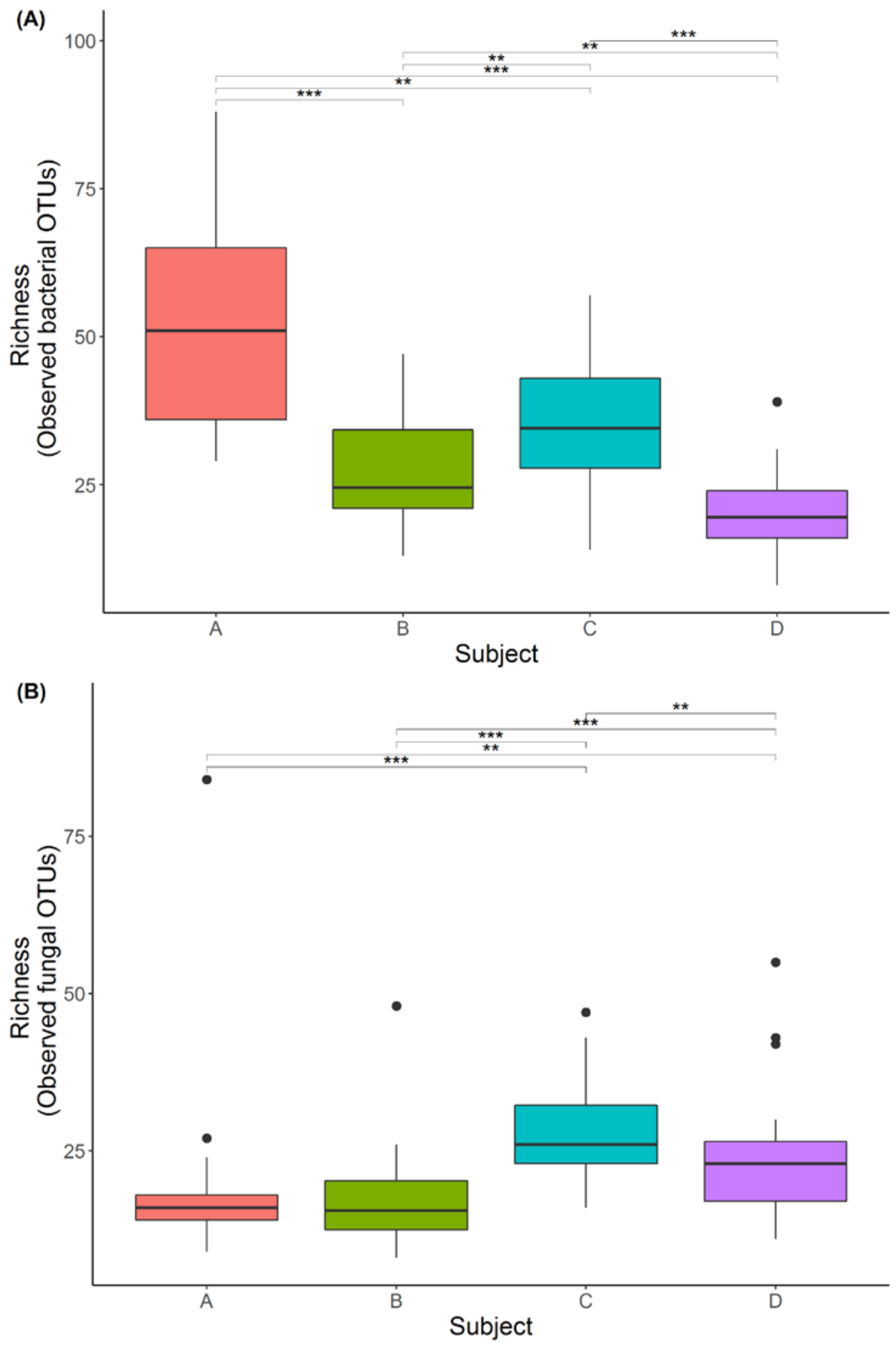

Figure S2. Box plots depicting alpha diversity as number of observed (A) bacterial or (B) fungal OTUs at 97% sequence similarity from middle meatus swab samples of the four subjects (A, B, C, D) included in this longitudinal study. Median values are indicated by the solid line within each box, extending to upper and lower quartile values. Outlier data points are indicated as closed black circles. Subject A harboured the most diverse bacterial community when compared to the other three subjects (50.93 OTUs ± 16.16 standard deviation, $p < 0.05$) and Subject D harboured a significantly less diverse bacterial community when compared to all other subjects (20.45 ± 6.00, $p < 0.05$). Subject C harboured the most diverse fungal community when compared all other subjects (27.73 ± 7.73, $p < 0.05$). * = p<0.05, ** = p<0.01, *** = p <0.001.

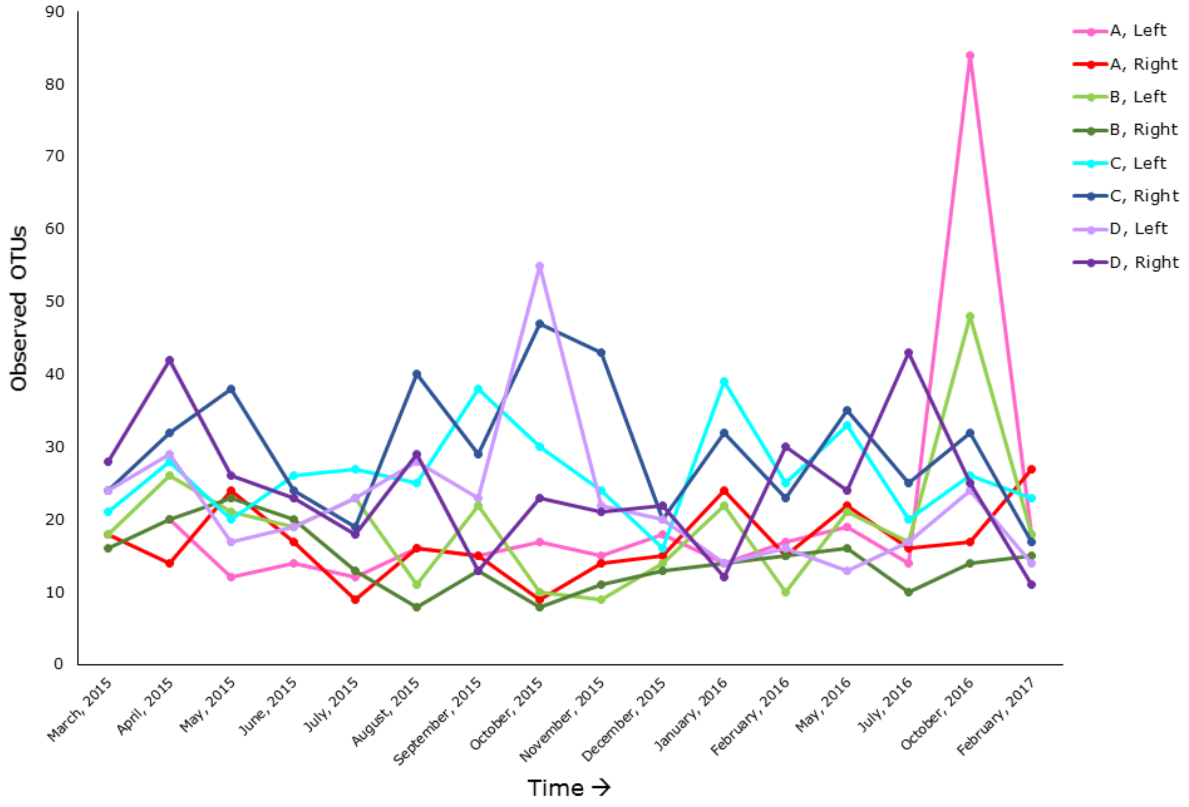

Figure S3. Number of observed fungal OTUs at 97% sequence similarity from the left and right sinuses of each individual throughout the two-year time period visualised in a line-graph.

(A)

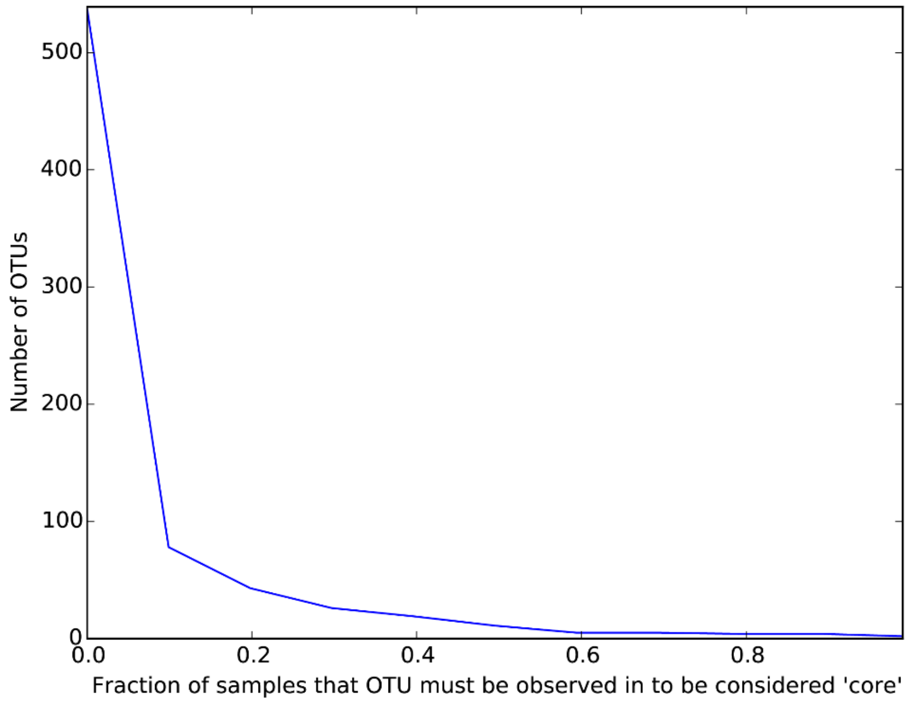

(B)

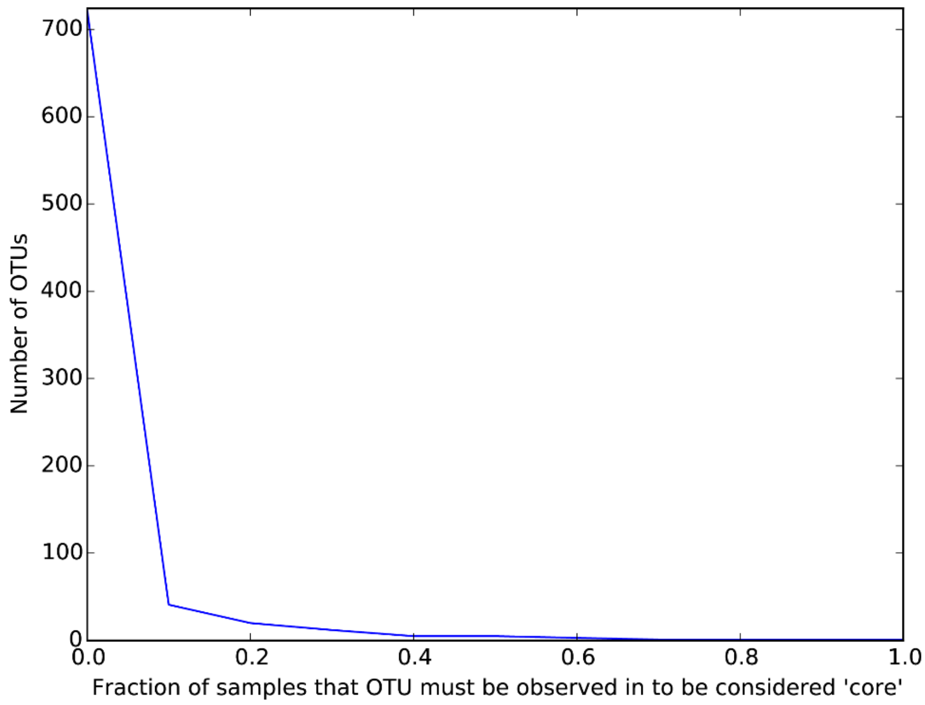

Figure S4. Line graphs depicting the number of (A) bacterial and (B) fungal OTUs that must be present to be considered part of the transient (< 20%), persistent (45-89%) or core community (90-100%) across the fraction of samples.

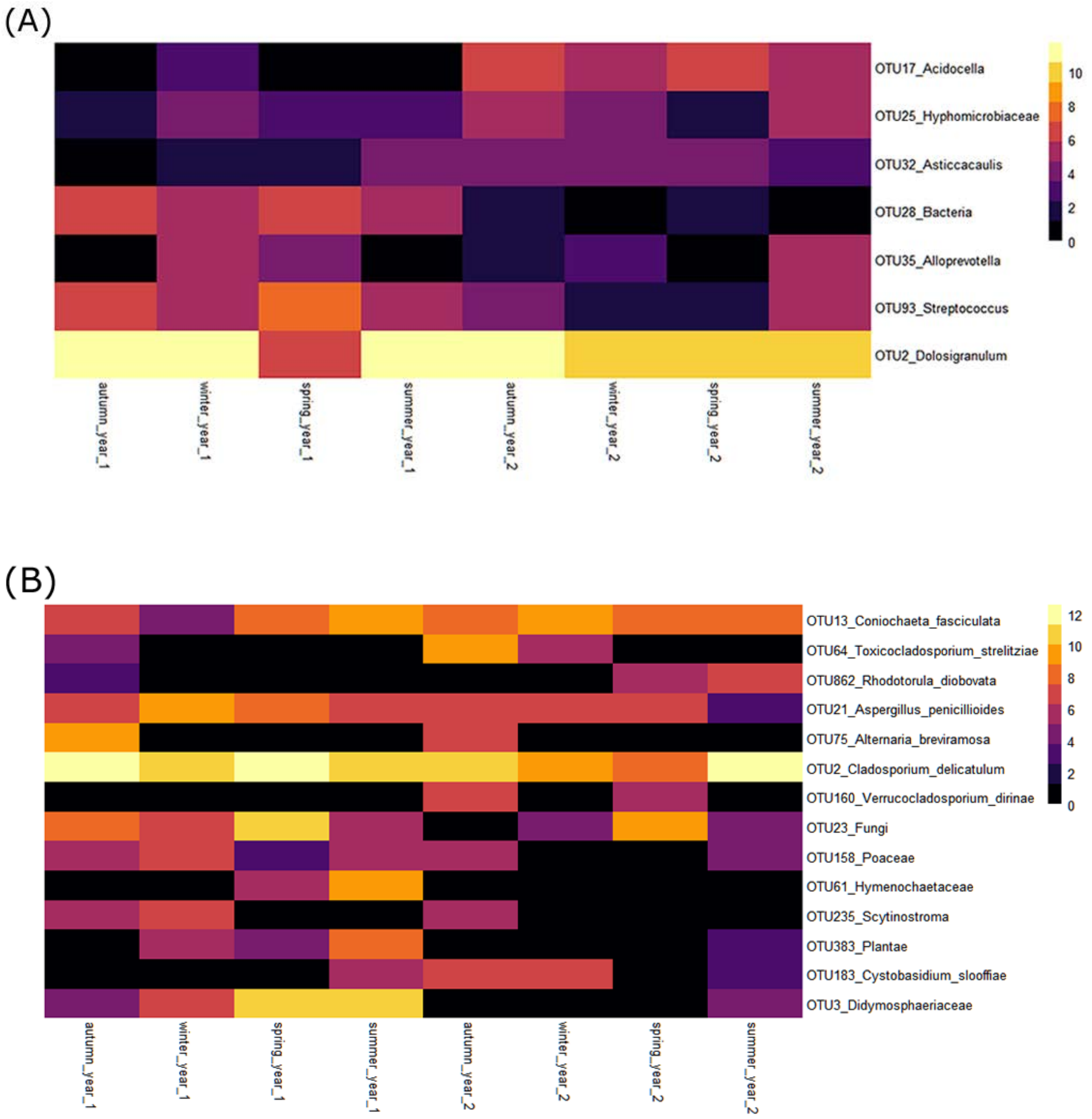

Figure S5. Heat maps depicting changes in relative abundances for those (A) bacterial and (B) fungal OTUs which shift significantly throughout the two-year sampling period. Relative abundances are $\log_2(x+1)$-transformed total sums.

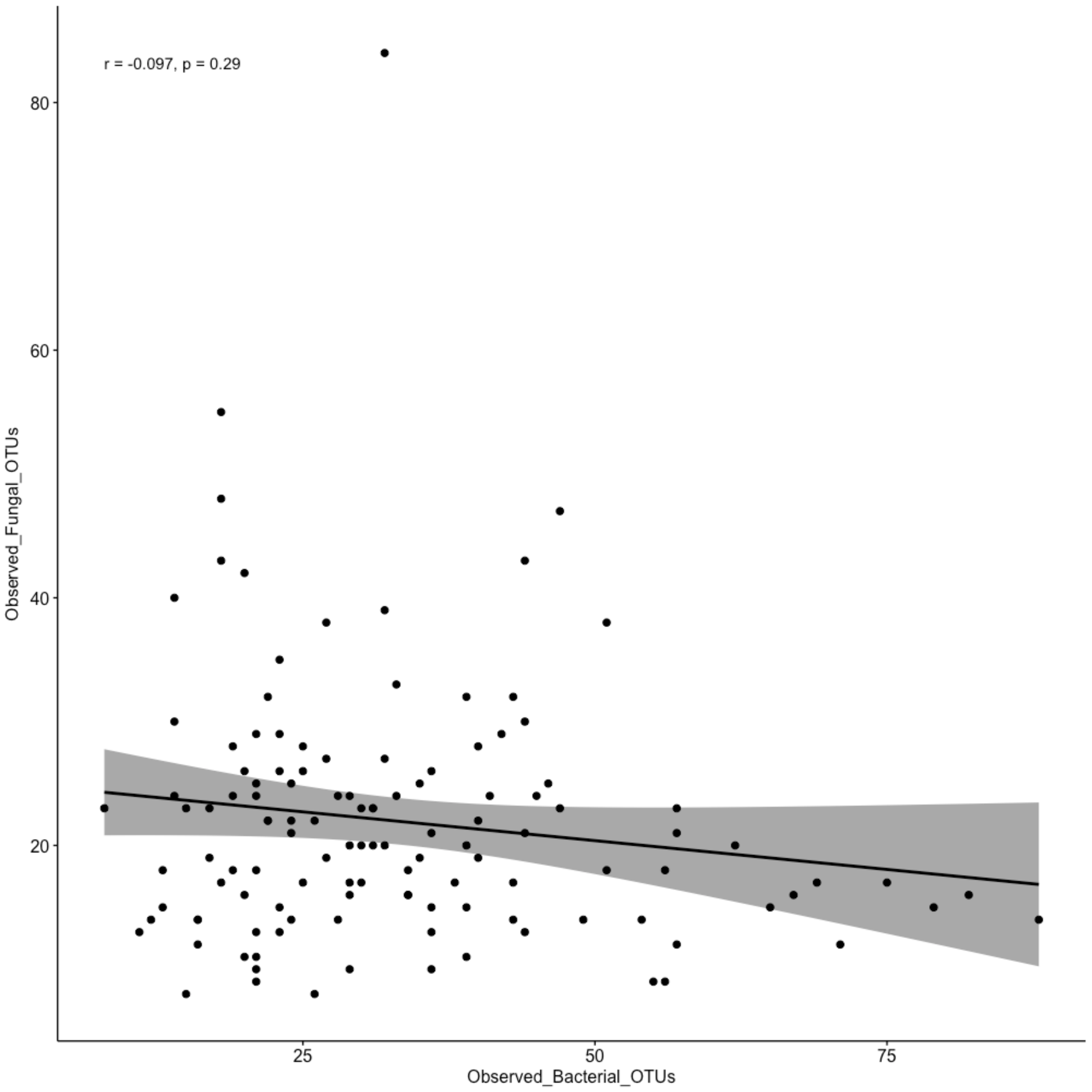

Figure S6. Scatterplot depicting the Spearman correlation between the numbers of observed bacterial and fungal OTUs across all samples. A weak, non-significant negative association is observed by the regression line (r = -0.097, $p > 0.05$). Positive and negative confidence intervals are represented by the shaded areas above and below the regression line, respectively.
